# Heart Failure with Preserved Ejection Fraction Correlates with Fibrotic Atrial Myopathy in Patients Undergoing Atrial Fibrillation Ablation

**DOI:** 10.3390/jcm13195685

**Published:** 2024-09-24

**Authors:** Jonghui Lee, Michael Sponder, Stefan Stojkovic, Martin Riesenhuber, Andreas Hammer, Thomas M. Hofbauer, Patrick Sulzgruber, Stefanie Burger, Stefan Kastl, Franz Duca, Robert Schönbauer

**Affiliations:** Department of Internal Medicine II, Division of Cardiology, Medical University of Vienna, 1090 Vienna, Austria

**Keywords:** HFpEF, atrial fibrillation, atrial myopathy

## Abstract

**Background:** The incidence of atrial fibrillation (AF) in patients with heart failure with preserved ejection fraction (HFpEF) is high. Impaired left atrial (LA) function is a major determinant in HFpEF. However, the extent of electrical LA tissue degeneration in HFpEF is unknown. Therefore, we sought to investigate the amount of arrhythmogenic and fibrotic LA tissue degeneration in HFpEF patients presenting for AF ablation. **Methods:** We prospectively screened consecutive patients presenting for first time AF ablation. The HFA-PEFF score was used to identify HFpEF patients. Bipolar high-density voltage mapping was created in sinus rhythm prior to ablation to evaluate the general LA bipolar voltage and quantify areas of low voltage. LVAs were defined as areas with bipolar voltage < 0.5 mV. **Results:** In total, 187 patients were prospectively enrolled (age 65 ± 11 years, 45% female, 46% persistent AF, 25% HFpEF) in this study. HFpEF patients were older and had a higher CHA2DS2-VASc score (70 ± 9 vs. 63 ± 11 years and 3.2 ± 1.5 vs. 2.3 ± 1.5, each *p* < 0.001, respectively). Overall, low-voltage areas (LVAs) were present in 97 patients (52%), whereas 76% of the HFpEF population had LVA, as compared to 44% of patients without HFpEF (*p* < 0.001). HFpEF was associated with generally decreased LA bipolar voltage (1.09 ± 0.64 vs. 1.83 ± 0.91 mV; *p* < 0.001) and predictive of the presence of low-voltage areas (76% vs. 44% *p* < 0.001). The HFA-PEFF score inversely correlated with LA bipolar voltage (=−0.454; *p* < 0.001). **Conclusions:** HFpEF closely relates to generally decreased LA bipolar voltage and to the existence of fibrotic and arrhythmogenic LA tissue degeneration.

## 1. Introduction

Atrial fibrillation (AF) and heart failure with preserved ejection fraction (HFpEF) are global epidemics, affecting millions of people worldwide [1]. As AF and HFpEF share many clinical features, such as arterial hypertension, overweight/obesity, and advanced age, it is not surprising that these two diseases frequently coexist. Up to 65% of the HFpEF population additionally suffer from AF. Conversely, up to 20% of AF patients are also diagnosed with HFpEF [2]. One main determinant that AF and HFpEF have in common is left atrial (LA) remodeling.

Impaired LA function is a common finding in both HFpEF and AF. But also, in various other cardiomyopathies such as cardiac amyloidosis and hypertrophic cardiomyopathy, impaired LA function plays a crucial role. Therefore, LA strain analysis as quantified by echocardiography can be a valuable adjunctive tool for diagnosing not only HFpEF, but also cardiac amyloidosis and hypertrophic cardiomyopathy [3,4].

If echocardiographic assessment leaves us with diagnostic uncertainties, cardiac magnetic resonance imaging (CMR) can serve as a very useful tool for ensuring the right diagnosis and also for further cardiovascular risk assessment in HFpEF and hypertrophic cardiomyopathies [5,6]. Besides that, CMR also provides a very suitable method for assessing phasic LA volumes. In addition, with the use of feature tracking algorithms, LA wall deformation and subsequently phasic LA function can be assessed with high accuracy [7].

For HFpEF patients, impaired LA function is an independent predictor for cardiovascular outcome [8,9]. In patients with symptomatic AF presenting for AF ablation, impaired LA function, as quantified by CMR, closely correlates with fibrotic and arrhythmogenic tissue degeneration [10]. Furthermore, LA fibrosis is a well-known predictor of AF recurrence after ablation [11]. Recent prospective randomized findings suggest a better outcome regarding freedom from arrhythmia recurrence if these fibrotic areas are targeted by ablation [12], on top of pulmonary vein isolation, which still remains the cornerstone of AF ablation [13]. Therefore, it is of great interest to know whether HFpEF patients present with a higher extent of LA fibrosis.

To date, there have been no data on the extent of fibrotic and arrhythmogenic LA remodeling in HFpEF patients undergoing AF ablation. The aim of this study was to elucidate the interference between HFpEF, AF, and low-voltage areas (LVAs) in patients presenting for first AF ablation. Therefore, we performed extensive LA bipolar voltage mapping to detect areas of low voltage, a widely accepted surrogate for fibrotic changes in HFpEF vs. non-HFpEF patients presenting for AF ablation [14].

## 2. Materials and Methods

### 2.1. Trial Design and Study Participants

This study is a single-center, prospective trial and was conducted at the Medical University of Vienna (Austria). The study was approved by the ethics committee of the Medical University of Vienna (EK Nr. 1295/2020).

Between November 2020 and July 2023, two hundred and thirteen consecutive patients presenting AF ablation for the first time using a 3D electroanatomic mapping system were screened for inclusion in the study. Study participants were eligible for inclusion if they had symptomatic paroxysmal or persistent ECG-documented AF, presenting for their first AF ablation procedure. Patients were excluded when one of the following exclusion criteria was met: pregnancy, acute infection, ongoing oncological history, non-cardiac liver cirrhosis, osteoporosis, known dementia, cardiac surgery, heart failure with reduced ejection fraction, or cardiac amyloidosis. All patients provided written informed consent prior to ablation. Thirteen patients were unwilling to participate, twelve patients had a left ventricular ejection fraction <40% and were therefore excluded, and one patient was also excluded from the study due to a diagnosis of cardiac amyloidosis. Screening for amyloidosis was performed according to current recommendations [15,16], including transthoracic echocardiography, cardiac magnetic resonance imaging, ^99m^Tc-3,3-diphosphono-1,2-propanodicarboxylic acid scintigraphy, and if necessary, endomyocardial biopsy. Therefore, 187 patients were prospectively included in the final study population. All included patients underwent baseline clinical assessment, laboratory testing, transthoracic echocardiography, and high-density bipolar voltage mapping.

### 2.2. HFA-PEFF Score

The diagnosis of HFpEF remains difficult, especially when AF coexists as both share common symptoms and risk factors [2]. Currently, there are two widely accepted diagnostic algorithms for the diagnosis of HFpEF, the HFA-PEFF score and H2FPEF score [17]. As the presence of AF is one parameter of the H2FPEF score but not of the HFA-PEFF score, we preferred the use of the HFA-PEFF score, as all of our possible study participants were patients presenting for AF ablation.

The HFA-PEFF score [17] was used to identify HFpEF patients among those presenting AF ablation for the first time. The HFA-PEFF consists of major and minor criteria with a major criterium accounting for 2 points and a minor criterium for 1 point. The score can be divided into three categories: functional, morphological, and biomarkers in sinus rhythm and in AF. Echocardiographic measurements and N-terminal prohormone of brain natriuretic peptide (NT-proBNP) levels are important parameters when using the HFA-PEFF score. A score of ≥5 points is considered a definite diagnosis of HFpEF.

### 2.3. High-Density Bipolar Voltage Map

All study participants underwent high-density bipolar voltage mapping of the LA at the beginning of the electrophysiological procedure, prior to AF ablation using a commercially available electroanatomic mapping system (CARTO3, Biosense Webster, Inc., Diamond Bar, CA, USA) to identify areas of impaired bipolar voltage. The mapping was performed in sinus rhythm, while pacing from the proximal bipole of a decapolar mapping catheter (InquiryTM Steerable Diagnostic Catheter, Abbott, IL, USA) introduced in the coronary sinus with a pacing cycle length of 600 ms. If patients were in AF, then extracorporal electrical cardioversion was used to convert them to sinus rhythm. Electroanatomic 3D reconstruction and bipolar voltage mapping was carried out simultaneously using a multispline, multipolar mapping catheter (CARTO™ PENTARAY™ NAV eco, Biosense Webster, Inc., Diamond Bar, CA, USA) in combination with an auto-annotation algorithm (CARTO, Biosense Webster, Inc., Diamond Bar, CA, USA) [10]. Before further assessment, all pulmonary veins were removed from the created 3D model at the level of their ostia. LVAs were defined as regions with bipolar voltage below 0.5 mV, a widely accepted cut-off value to define fibrotic and arrhythmogenic areas [14,18]. The LA was further divided into anterior, posterior, septal, left-lateral, roof, and inferior regions. The mean voltage (mV) and the extent of LVAs in relation to total LA surface area (%) were also calculated.

### 2.4. Statistical Analysis

Descriptive statistics were applied to describe the study population and procedural specifics. Results were summarized with numbers and percentages for categorical variables and means ± standard deviation for continuous variables. Dichotomous variables were compared using the chi-squared test; for continuous variables, the Mann–Whitney U test was applied. Metric variables were illustrated by boxplots. Possible correlations were assessed using the Spearman rank correlation coefficient. Statistical analyses were conducted with IBM SPSS Version 29. A (two-tailored) *p*-value below 0.05 indicated statistical significance for all tests.

## 3. Results

### 3.1. Baseline Characteristics

Table 1 summarizes the baseline characteristics of 47 patients with definite diagnosis of HFpEF (HFA-PEFF score ≥5) versus 140 patients without HFpEF (HFA-PEFF score < 5). Patients with definite HFpEF were older (*p* < 0.001) and more likely to have a history of renal failure (*p* = 0043), a higher CHA2DS2-VASc score (*p* < 0.001), and higher NTproBNP levels in sinus rhythm (*p* < 0.001) and AF (*p* = 0.001). Moreover, LA volume index was higher in HFpEF patients as compared to patients without HFpEF (*p* < 0.001).

### 3.2. Electrophysiological Findings

**Figure 1:**
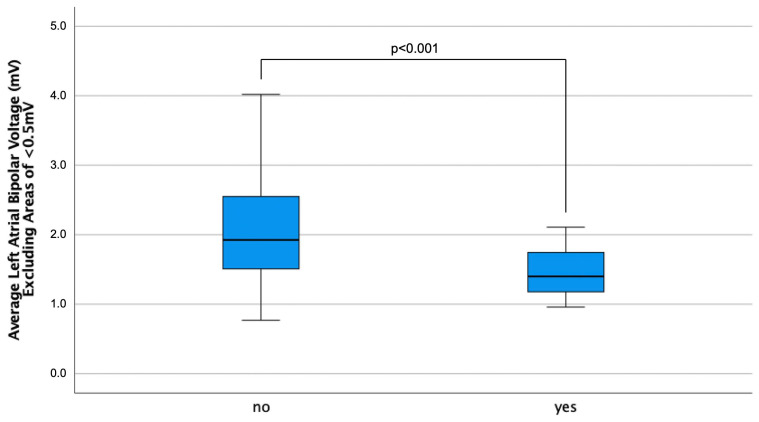
Boxplots displaying the average left atrial bipolar voltage amplitude, excluding areas of <0.5 mV, according to the presence/absence of HFpEF.

Table 2 summarizes the electrophysiological findings of the mapping procedure. HFpEF patients had a larger LA surface area (138 ± 40 vs. 167 ± 42 cm^2^, *p* < 0.001) and correspondingly more mapping points (1258 ± 719 vs. 1685 ± 818, *p* < 0.001). In total, 76% of patients with a definite HFpEF diagnosis versus 44% of controls had areas of low voltage (<0.5 mV) (*p* < 0.001). Notably, in the population with the presence of low-voltage areas, there was no significant difference in the size of low-voltage areas in definite HFpEF versus no HFpEF. The average bipolar LA voltage was significantly decreased (*p* < 0.001). Interestingly, this significant difference was also present when only LA bipolar voltage levels of >0.5 mV were compared (Figure 1).

**Figure 2:**
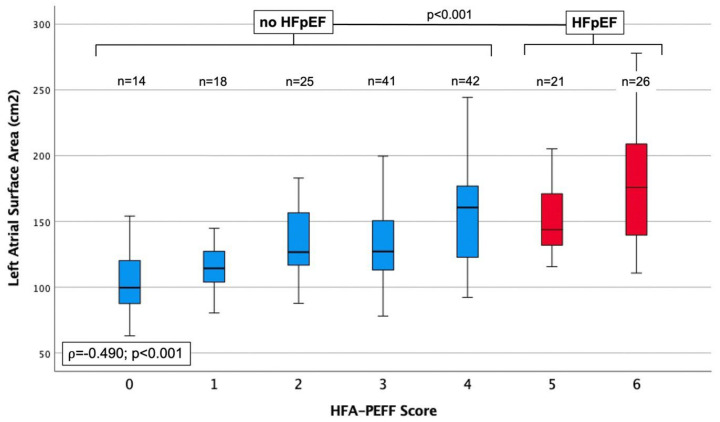
Boxplots displaying the left atrial surface area, according to ascending values of the HFA-PEFF score.

**Figure 3:**
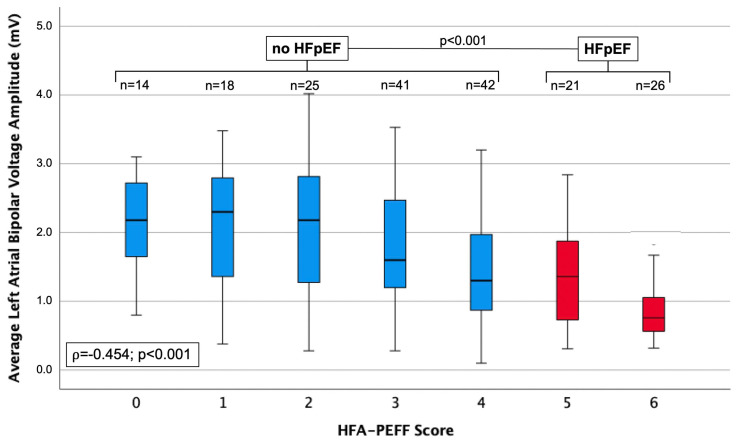
Boxplots displaying the average left atrial bipolar voltage amplitude, according to ascending values of the HFA-PEFF score.

This finding demonstrates that the significantly lower LA bipolar voltage level is not driven by the higher incidence of low-voltage areas in the HFpEF population. Table 3 summarizes the electrophysiological findings in relation to the HFA-PEFF score. There was a positive correlation between the HFA-PEFF score and LA surface area (Figure 2), with an increasing presence of low-voltage areas and decreasing levels of bipolar voltage ≥0.5 mV (Figure 3) (each *p* < 0.001).

## 4. Discussion

### 4.1. Main Findings

To date, this is the first study to evaluate the extent of electrical LA myopathy using high-density bipolar voltage maps in HFpEF patients undergoing first-time AF ablation. 

This study has three main findings:

- The presence of LA low-voltage areas is significantly associated with the diagnosis of HFpEF;
- Besides the presence of low-voltage areas, HFpEF patients show a generally decreased LA bipolar voltage;
- HFpEF is associated with a larger LA surface area.

**Figure 4:**
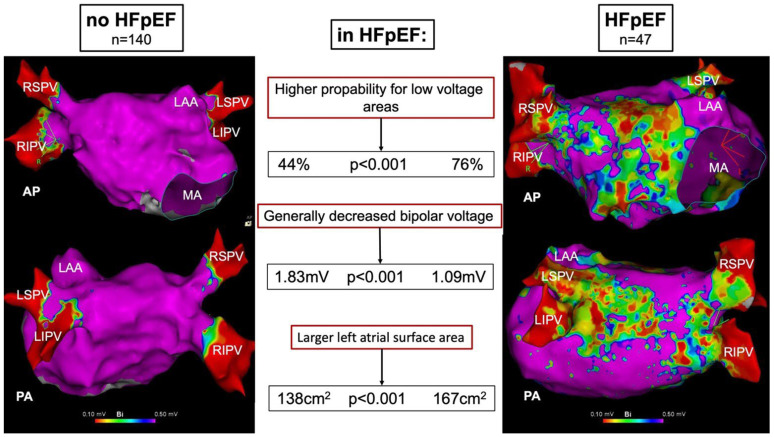
Direct comparison of a high-density left atrial voltage map of a patient without (HFA-PEFF score 0) vs. definite HFpEF (HFA-PEFF score 6). Low-voltage areas are defined as <0.5 mV. Patients with HFpEF have a significantly higher likelihood for the presence of low-voltage areas. Besides the presence of low-voltage areas, HFpEF patients show a generally decreased LA bipolar voltage and larger left atrial surface area. HFpEF, heart failure with preserved ejection fraction; AP, anterior–posterior; LAA, left atrial appendage; LIPV, left inferior pulmonary vein; LSPV, left superior pulmonary vein; PA, posterior–anterior; RIPV, right inferior pulmonary vein; RSPV, right superior pulmonary vein.

Figure 4 summarizes the main findings.

### 4.2. Prevalence of Low-Voltage Areas

A representative study investigated the prevalence and predictors of low-voltage areas in patients presenting for AF ablation [18]. Similar to our study, bipolar voltage mapping was performed and zones with bipolar voltage of <0.5 mV were defined as low-voltage areas. The authors found an overall incidence of patients with low-voltage areas of 35%, which is lower than the proportion of 52% of patients with low-voltage areas of our study population. An explanation for this difference can be found in the baseline characteristics of the different study populations. For example, the mean CHADSVASc score, which also serves as a predictor for the existence of low-voltage areas [11], of their study population was 2.3, compared to 2.6 of our collective. Another well-known predictor for low-voltage areas is LA enlargement and increased LA surface area [18,19]. The median LA surface area of their study population was 103 cm^2^, which was significantly lower than the 133 cm^2^ of our study collective.

### 4.3. Atrial Fibrillation Ablation in HFpEF

So far, only a few prospective randomized clinical trials and subsequent meta-analyses have demonstrated a benefit in mortality for patients with heart failure with reduced ejection fraction undergoing AF ablation when compared with non-invasive treatment [20]. For HFpEF patients undergoing AF ablation, prospective randomized clinical data regarding the mortality outcome are still missing. However, the CABA-HFpEF trial is currently addressing this issue (NCT05508256).

Regarding surrogate parameters, a small study investigating invasive hemodynamics before and after AF ablation in a well-defined HFpEF collective of 20 participants showed a significant decrease in pulmonary capillary wedge pressure below the threshold for HFpEF diagnosis, in those patients without arrhythmia recurrence. Notably, 11 of the 20 patients had arrhythmia recurrence [21].

A number of observational trials have shown partly conflicting results regarding the prognostic benefit of AF ablation in HFpEF on cardiovascular outcomes. For example, a post hoc analysis of the largest prospective randomized AF ablation CABANA trial has shown a mortality benefit in the sub-population of 580 patients suffering from HFpEF [22]. However, it should be noted that this analysis was performed by imputing missing left ventricular ejection fraction values; without this imputation, there was no survival benefit. A large-scale retrospective propensity-matched study including >16.000 HFpEF patients with AF, of whom >1.000 underwent AF ablation, showed no mortality benefit of AF ablation [23]. On the other hand, a just recently published retrospective propensity-matched cohort study showed a significant reduction in the primary endpoint, which was a composite of all-cause death and rehospitalization for worsening of heart failure, in HFpEF patients undergoing AF ablation, compared with a conservative medical treatment control group. Notably, the positive trial results were mainly driven by a reduction in hospitalization for worsening of heart failure [24].

Due to these contradictory results, prospective randomized data are paramount to better assess the potential positive effect of AF ablation in HFpEF on morbidity and mortality.

### 4.4. The Prognostic Importance of Atrial Myopathy

Impaired LA function is a hallmark feature of patients suffering from HFpEF [25,26]. However, the extent of electrical disease in HFpEF is unknown. Our study shows for the first time that patients with HFpEF and AF display a generally decreased LA bipolar voltage and more frequently show areas of fibrotic LA tissue degeneration, when compared to patients without HFpEF. In addition, decreased LA bipolar voltage and the presence of areas of fibrotic LA tissue degeneration correlated well with an increasing HFA-PEFF score. This finding is of particular interest as LA scarring is a major cause of AF recurrence after pulmonary vein isolation [11] and may explain the conflicting results of observational studies regarding AF ablation in HFpEF.

## 5. Clinical Implications

It should be of great interest for cardiac electrophysiologists that HFpEF patients undergoing AF ablation are more likely to have arrhythmogenic and fibrotic LA tissue degeneration, which is a risk factor for AF recurrence after pulmonary vein isolation. Prospective randomized data suggest a better outcome in terms of reduced AF burden after ablation if these arrhythmogenic areas are targeted by ablation in addition to pulmonary vein isolation [12].

However, ablation procedures with additional substrate modification on top of pulmonary vein isolation do require significantly longer procedure times. For better procedure planning, pre-procedural imaging can be used to assess the likelihood of the presence of areas of fibrotic tissue degeneration [10].

On the other hand, our data also show a more advanced electrical LA myopathy with a higher likelihood of the presence of LVAs and a generally decreasing LA bipolar voltage with an increasing HFA-PEFF score. These findings may suggest a further progression of fibrotic and arrhythmogenic LA myopathy according to advanced HFpEF stages. Accordingly, AF ablation would be more rational in earlier stages of HFpEF, to have a higher chance of maintaining SR after ablation, which in turn has the potential of reversing HFpEF typical hemodynamic changes [21]. Similarly, in the ongoing prospective, randomized CABA-HFpEF trial, a history of AF of more than two years is an exclusion criterion to avoid the inclusion of patients with advanced stages of HFpEF (NCT05508256).

## 6. Limitations

This was a single-center study; thus, a center-specific bias cannot be ruled out and study results may not be generalized. On the other hand, single-center studies also have some advantages, such as homogenous patient selection and continuous workflow. HFpEF diagnosis was solely based on an HFA-PEFF score ≥5. In case of an HFA-PEFF score of 2–4, a further diagnostic approach with right heart catheterization or stress echocardiography is recommended for definite diagnosis or ruling out of HFpEF [10], which we did not perform in our study. Therefore, we might have missed some HFpEF diagnoses within our study population. However, we could show a continuous decline in average LA bipolar voltage according to an ascending HFA-PEFF score (ρ = −0.454; *p* < 0.001) (Figure 3).

## 7. Conclusions

Patients who suffer from HFpEF and concomitant AF do have significantly higher chances for arrhythmogenic and fibrotic LA myopathy, as defined by decreased bipolar voltage and the presence of LVAs. This fact is of major interest, since LA scarring, if not targeted by ablation, is a risk factor for AF recurrence after pulmonary vein isolation. Further preprocedural imaging may be helpful in identifying these patients.

## Figures and Tables

**Table 1:** Baseline characteristics.

Variable	HFpEF No (n = 140)	Yes (n = 47)	*p*-Value
Age, y	63 ± 11	70 ± 9	**<0.001**
Female sex, n (%)	58 (41)	27 (57)	0.064
Body mass index, kg/m^2^	27 ± 5	28 ± 5	0.984
Persistent AF, n (%)	62 (44)	24 (51)	0.230
Arterial hypertension, n (%)	90 (64)	30 (64)	1.000
Renal failure, n (%)	4 (3)	5 (11)	**0.043**
Diabetes mellitus type II, n (%)	23 (16)	8 (17)	1.000
Cardiovascular disease, n (%)	41 (29)	17 (36)	0.361
Hyperlipidemia, n (%)	67 (48)	18 (38)	0.313
Stroke, n (%)	16 (11)	3 (6)	0.414
CHA_2_DS_2_-VASc score	2.3 ± 1.5	3.2 ± 1.5	**<0.001**
NT-proBNP, ng/mL (SR)	354 ± 572	638 ± 526	**<0.001**
NT-proBNP, ng/mL (AF)	1193 ± 968	1915 ± 1382	**0.001**
LAVI, mL/m^2^	31 ± 14	45 ± 15	**<0.001**
Interventricular Septum, mm	12 ± 2.1	12.6 ± 2.4	0.696
HMG-CoA reductase inhibitor, n (%)	66 (47)	20 (43)	0.501
Betablocker, n (%)	105 (75)	37 (79)	0.550
Diuretics, n (%)	51 (36)	26 (55)	**0.024**
ACE inhibitor, n (%)	23 (16)	11 (23)	0.275
AT II receptor antagonist, n (%)	46 (33)	21 (45)	0.156

Values are given as mean ± standard deviation or total numbers and percent. HFpEF = heart failure with preserved ejection fraction; AF = atrial fibrillation; NT-proBNP = N-terminal prohormone of brain natriuretic peptide; LAVI = left atrial volume index; HMG-CoA = 3-hydroxy-3-methyl-glutaryl-coenzyme A; ACE = angiotensin-converting enzyme; AT II = angiotensin II. Bold *p*-values indicate statistically significant differences.

**Table 2:** Electrophysiological mapping data.

Variable	HFpEF No (n = 140)	Yes (n = 47)	*p*-Value
Mapping points	1258 ± 719	1685 ± 818	**<0.001**
LA surface area, cm^2^	138 ± 40	167 ± 42	**<0.001**
Bipolar voltage, mV	1.83 ± 0.91	1.09 ± 0.64	**<0.001**
Low-Voltage Area, n (%)	61 (44)	36 (76)	**<0.001**
Low-Voltage Area, cm^2^	29.5 ± 27.0	30.2 ± 24.5	0.786
Low-Voltage Area, % of LA surface Area	19.0 ± 14.3	23.5 ± 15.8	0.163
Bipolar Voltage >0.5 mV, mV	2.04 ± 0.70	1.51 ± 0.45	**<0.001**

Values are given as mean ± standard deviation; LA = left atrial. Bold *p*-values indicate statistically significant differences.

**Table 3:** Electrophysiological findings in relation to the ascending HFA-PEFF score.

HFA-PEFF SCORE	0	1	2	3	4	5	6		
								**ρ**	***p*-Value**
**N**	14	18	25	41	42	21	26		
**LA SURFACE AREA, cm^2^**	110 ± 35	115 ± 19	134 ± 26	139 ± 39	159 ± 44	153 ± 32	180 ± 46	**0.490**	**<0.001**
**BIPOLAR VOLTAGE, mV**	2.21 ± 0.95	2.13 ± 0.98	2.09 ± 1.02	1.79 ± 0.80	1.43 ± 0.78	1.35 ± 0.74	0.86 ± 0.42	**−0.454**	**<0.001**
**LVA, N (%)**	3 (21)	6 (33)	5 (20)	19 (46)	28 (67)	13 (62)	23 (88)	**0.418**	**<0.001**
**LVA, cm^2^**	21.2 ± 9.9	30.7 ± 16.6	28.1 ± 12.6	27.0 ± 27.5	31.9 ± 32.1	22.1 ± 26.2	35.7 ± 22.2	0.070	0.527
**LVA, % OF LA SURFACE AREA**	15.2 ± 11.2	23.2 ± 11.6	23.0 ± 11.3	17.4 ± 17.1	18.7 ± 14.2	24.2 ± 19.3	23.0 ± 13.5	0.101	0.358
**BIPOLAR VOLTAGE > 0.5 mV, mV**	2.35 ± 0.80	2.30 ± 0.75	2.21 ± 0.85	2.05 ± 0.56	1.72 ± 0.56	1.67 ± 0.54	1.37 ± 0.29	**−0.450**	**<0.001**

Values are given as mean ± standard deviation; LA = left atrial; LVA = low-voltage area. Bold *p*-values indicate statistically significant differences.

## Data Availability

The data presented in this study are available on request from the corresponding author.
